# Episodic future thinking decreases cocaine consumption among individuals with cocaine use disorder

**DOI:** 10.21203/rs.3.rs-10695291/v1

**Published:** 2026-09-03

**Authors:** Rafaela Fontes, Allison Tegge, Samuel McClure, Mikhail Koffarnus, Jeffrey Soldate, Anita Kablinger, Warren Bickel, Stephen LaConte

**Affiliations:** Fralin Biomedical Research Institute

## Abstract

Psychosocial treatments remain the standard approach for cocaine use disorder (CUD). However, high dropout rates and a lack of significant response to these treatments are commonly observed, highlighting the need for novel, scalable interventions to support recovery from CUD. Episodic future thinking (EFT) is a scalable intervention that has been shown to decrease delay (DD) discounting and cocaine valuation after a single exposure among individuals with CUD. Expanding on those findings, we conducted a pilot clinical trial in a CUD sample to evaluate the: 1) feasibility of prolonged EFT exposure and remote monitoring of cocaine consumption; 2) efficacy of prolonged exposure to EFT in decreasing DD and cocaine consumption; and 3) efficacy of prolonged exposure to EFT in decreasing hypothetical demand for cocaine. Participants were randomized to EFT (n = 41) or control episodic thinking (n = 39) and completed a five-week study, during which they completed four cue generation sessions, received cue reminders twice daily, and reported daily cocaine consumption. Results indicated that prolonged exposure to daily EFT reminders and daily consumption reporting was feasible, with more than 70% of participants in both groups completing the entire study and daily submissions above 80% for both groups. Consistent with our hypotheses, significant decreases in DD and amount of cocaine consumption were observed for participants in the EFT group, but not in CET. However, contrary to our hypothesis, robust effects of EFT on hypothetical demand were not observed.

## Introduction

Cocaine use disorder (CUD) significantly contributes to disability, morbidity, and mortality^1–4^. In the US, overdose by cocaine nearly tripled from 2013 to 2018^5^ and continued to increase in 2023^6^. With the lack of FDA-approved pharmacological interventions for CUD, psychosocial treatments remain the standard approach. Interventions such as intensive outpatient therapy, contingency management, and cognitive-behavioral therapy are among the most common and effective CUD treatments^7^. However, high dropout rates and a lack of significant response to these treatments are commonly observed^8,9^. Furthermore, the frequency and intensity required for some interventions to be effective, and the cost of some of these treatments, complicate the scalability and reach of the target population^7^. Therefore, there is a considerable need for novel, scalable interventions for CUD.

One scalable intervention that has been shown effective in decreasing substance valuation and consumption is episodic future thinking (EFT)^10^. EFT is an intervention based on the science of prospection, during which participants are prompted to describe in vivid detail positive future events they look forward to experiencing^11,12^. The use of EFT as an intervention for substance use disorders is informed by the Reinforcer Pathology (RP) theory^13–15^. Central to RP is the notion that reinforcer value is integrated over a temporal window, which can be measured through delay discounting (DD). Short temporal windows (i.e., high DD) increase the relative value of short, brief, intense, and immediate reinforcers, such as cocaine, over reinforcers that accrue value over time, such as long-term health. Conversely, long temporal windows reverse these relative valuations. Indeed, empirical evidence suggests that high DD is ubiquitous in substance use disorders^16–18^. Moreover, DD has been suggested to serve as a behavioral marker at all stages of the addiction process^19^ and has been predictive of therapeutic outcomes^20–22^. Thus, according to RP, substance use disorder is associated with short temporal windows, which results in higher relative valuation of immediate and intense reinforcers, including cocaine. EFT is an intervention that works by extending one’s temporal window, thereby decreasing the relative value associated with substance use, and has been shown to decrease DD and substance valuation in several substance use disorders^10^.

Previous studies have shown that a single exposure to EFT decreased DD^23,24^ and cocaine valuation in a hypothetical task^24^ among individuals with CUD. However, whether EFT is effective in decreasing real-world cocaine consumption remains unknown. Furthermore, no prior studies have explored the effects of multiple EFT intervention sessions and continued exposure to daily EFT on discounting, consumption, or behavioral economic demand among individuals with CUD. Therefore, we conducted a pilot clinical trial to evaluate the: 1) feasibility of prolonged EFT exposure and remote monitoring of cocaine consumption; 2) efficacy of prolonged exposure to EFT in decreasing DD and cocaine consumption; and 3) efficacy of prolonged exposure to EFT in decreasing hypothetical demand for cocaine.

## Methods

This was a parallel trial comparing active (EFT) and control conditions. Participation in the study lasted about five weeks, during which participants completed a baseline (S1) and four intervention sessions (S2-S5) in the laboratory. Each session was comprised of behavioral assessments and urinalysis screening. During S1, S2, and S5, participants also completed an fMRI scan. Participants completed additional in-laboratory visits between intervention sessions, during which they underwent urinalysis screening and behavioral assessments for a secondary aim. Neuroimaging and secondary aims results will be reported elsewhere. During the five weeks, participants also completed daily remote monitoring of cocaine consumption via text messages. Figure 1 shows the study timeline. Total compensation was approximately $1000 for completing all in-laboratory visits and remote submissions. All procedures were registered at ClinicalTrials.gov (NCT05507814) and received ethical approval from the institutional review board at Virginia Polytechnic Institute and State University (Protocol No. 22–529).

### Participants

Participants were recruited from the Roanoke, VA community via flyers posted in the community (e.g., bars, restaurants, grocery stores), word-of-mouth, electronic (e.g., Craigslist, Facebook), and bus advertisements. All advertisement materials noted that this was not a treatment study, and participants were not informed about the study aims. Eligibility to sign consent was assessed with an online pre-screening questionnaire, and included 1) be 18 years of age or older, 2) demonstrate recent cocaine use (i.e., self-report of cocaine use at least twice in last month) and meet DSM-5 criteria for CUD (i.e., 2 or more symptoms), 3) meet moderate to severe criteria for any SUD (cocaine or other), 4) have a desire to quit or cut down on their cocaine use, but no proximate plans to enroll in treatment for CUD during the study period. Participants were ineligible if they 1) met moderate to severe DSM-5 criteria for opioid use disorder and were not in treatment, 2) had a current unmanaged psychotic disorder, 3) had a history of seizure disorders or traumatic brain injury they had not fully recovered from, 4) had any contraindication for participation in the MRI sessions, or 5) reported current pregnancy or lactation. Eligibility to continue in the study was reassessed after a baseline remote-monitoring phase that occurred between S1 and S2 (Week 1). Only participants who reported cocaine use at least once during this baseline period were invited to continue in the study.

A power analysis assuming a medium effect size on DD, our therapeutic target (f = 0.25 based on previous studies^24^), a repeated-measures correlation of 0.5, a Type I error rate of 0.01, and 95% statistical power indicated that 76 participants were needed to complete this study (n = 38 per group). G*Power was used to estimate sample size^25^. We over-recruited because the longitudinal nature of the study, increased screening procedures, and its time demands could contribute to high dropout rates.

#### Interventions

Participants who reported at least one day of cocaine use during baseline were invited to continue in the study and randomized to EFT or control episodic thinking (CET) with an even allocation ratio. As done previously^26–29^, the randomization procedure used a computerized algorithm that adjusted the probability of group assignment and biased the allocation to balance the groups on baseline DD rates and sex. Participants in both groups underwent four interview-guided EFT/CET cue-generation sessions at S2, S3, S4, and S5. After S2, participants received their EFT/CET cues twice a day via text messages for four weeks (Weeks 2–5).

#### Cue Generation

During the interview-guided EFT/CET cue-generation sessions, participants in both groups were instructed to think vividly and describe in detail positive events for six time points. Participants were prompted to identify clear, easy-to-imagine events; positive experiences they would like to have; and events that did not involve substance use. The only difference between the groups was the time orientation for the events. The EFT group was prompted to think about positive events that would occur in the future (i.e., 1 week, 1 month, 3 months, 1 year, 5 years, and 25 years in the future). The CET group was prompted to think about positive events that occurred in the recent past (i.e., last night from 7pm-10pm, yesterday between 4pm-7pm, yesterday between 1pm-4pm, yesterday from 10am-12pm, yesterday between 7am-10am, and the night before last between 7pm-10pm). Participants were instructed to think about one event per time point. After identifying the relevant event for that time point, they were asked a series of standard questions to prompt them to think about the details of the event (e.g., where they are/were, who they are/were with, how they are/were feeling, etc.). Participants then provided a description of the event (long cue) and a short phrase to function as a reminder (short cue; see Supplemental Materials for examples of long and short cues).

#### Cue exposure

Participants received reminders^30,31^ about their EFT/CET events (i.e., short cues) through SMS text twice daily (at 10am and 4pm). The short cues were texted in a random order, and each cue was followed by one randomly selected question designed to increase engagement with the cue (see Supplemental Materials for list of questions). Participants received $1 for responding to each question.

#### Measurements

##### Delay Discounting.

During each session (S1-S5), participants completed an adjusting-amount DD task, where they chose between smaller-sooner and larger-later amounts of money. The task included six delays, ranging from 1 week to 25 years in the future, and six trials for each delay. The larger-later amount remained constant at $1000 across trials, and the smaller-sooner amount was titrated according to the participant’s previous choice, as described in Du et al.^32^. During all intervention sessions (S2-S5), participants’ short cues were displayed above each choice (see Supplemental Materials for example). For EFT, cues were matched to their corresponding delay (i.e., 1-week cue shown during 1-week delay choices). For CET, the cues corresponded to their proximity to the present (e.g., last night from 7pm-10pm cue shown during 1-week delay choices).

##### Cocaine purchase task (CPT).

During each session (S1-S5), participants completed the CPT where they were presented with 13 prices (one price per page) in ascending order^33^ and instructed to indicate how many grams of cocaine they would buy at each price. Prices ranged from $0.00 to $80/gram. Because CPT does not include a temporal component, participants in both groups saw the same cue throughout the task, as done previously^34^ (1 year cue for EFT, and yesterday from 10:00am to 1:00pm cue for CET; see Supplemental Materials for example). The CPT instructions and unit (from hits to grams) were changed midway through the trial because participants were struggling to understand the task and provide high-quality data. Thus, only a subset of the participants completed the final version of this task.

##### Cocaine Consumption.

During the entire study, participants were required to report their previous day cocaine consumption on a scale from 0 to 4 (0 = none, 1 = light, 2 = moderate, 3 = heavy, and 4 = maximum ever used) via text. Participants received a prompting text every day at 1pm asking them to report their previous day’s consumption. Previous-day, instead of same-day consumption, was preferred to best capture all cocaine consumed each day without inconsistent response times compromising accuracy of the measure. Participants received $1 for their consumption reporting, regardless of the results. If a participant had not reported their consumption by the time they received a new prompt, they were asked to report consumption for both the previous day and the day before. Payment for consumption reporting was received only for the previous day's consumption. Midway through the study, we also started asking participants to report the number of grams consumed the previous day. This allowed us to explore correlations between the scale and the cocaine amount in grams. The remote monitoring lasted approximately 35 days (see Fig. 1). Because the length of the monitoring phases was contingent on session completion (i.e., Week 1 ended at S2, Week 2 ended at S3, Week 4 ended at S4, and Week 5 ended after they completed their last in-laboratory visit approximately one week after S5), the phase durations varied across participants (e.g., due to participant or staff availability for the sessions). **Tables S1** and **S2** show mean and median length of each monitoring week per group.

##### Urinalysis.

During every lab visit (13 total, including the intervention sessions), participants provided a urine sample that was screened for the presence of ten substances (cocaine, amphetamines, methamphetamines, barbiturates, benzodiazepines, opiates, methadone, oxycodone, phencyclidine, and cannabinoids) using immunoassay strips. Only results for cocaine were analyzed. Samples that met the established cutoff concentration (300 ng/mL) were considered positive.

### Data Analysis

#### Data cleaning

Due to a scheduling error, one participant had only one day of baseline monitoring and was therefore excluded from the analyses. Six participants were partnered with other enrolled participants, but they were still simultaneously enrolled in the study to avoid attrition. To maintain blinding to the group assignment, those six participants were assigned to the same group as their partners. These six matched participants were excluded from the analyses (EFT = 3; CET = 3). An additional five participants were related to other enrolled participants (e.g., partner, relative, roommate) but enrolled in the study after the first of the pair had already completed the study. Those additional five were randomized rather than matched. However, we also excluded them from the final analyses to avoid dependence between observations (EFT = 3; CET = 2). The final analytical sample (i.e., complete case sample) included 80 participants (EFT = 41; CET = 39), except for the CPT analysis, which included 58 participants due to the changes in task format (EFT = 29; CET = 29). As an additional cleaning step, we excluded data from sessions in which the participant failed the attention check for the delay-discounting task. In those cases, the participant was maintained in the sample, and the deletion was made at the session level (n = 19 in S1; n = 10 in S2; n = 5 in S3; n = 9 in S4; n = 5 in S5).

#### Statistical Analyses

The Statistical Analysis Plan was pre-registered on the Open Science Framework (OSF) and can be found at https://osf.io/hkvp8/files/osfstorage/6904cbcbb8eeeea84c9c3ac8. Any deviations from the SAP are noted in this section. All analyses were conducted using R software (version 4.6). We performed both complete case and intention-to-treat analyses. For the complete case, only participants who completed all sessions and were not excluded during the cleaning procedure were included (EFT = 41, CET = 39). For the intention-to-treat analyses, all participants assigned to groups (including the matched participants and those excluded during data cleaning) were included (EFT = 59, CET = 60), regardless of study completion. For participants who did not complete the intervention, all sessions after discontinuation were treated as missing data, and all daily consumption submission data up to their last submission were included. All intention-to-treat analyses are included in the Supplemental Materials. For all statistical analyses, significance was set at *p*<.05. Although not specified in the SAP, all statistical models were adjusted for age, sex, education, income, and CUD to assess the robustness of the effects after controlling for covariates. Follow-up analyses for significant interactions were conducted using estimated marginal means to evaluate simple interaction contrasts. Specifically, simple interaction contrasts compared between-group differences in changes from baseline. Pairwise comparisons were also performed to examine changes from baseline within each group. Degrees of freedom were estimated using the Kenward-Roger method^35^. P-values were corrected for multiple comparisons using the Holm method for simple interaction contrasts and the Tukey method for pairwise comparisons.

Retention was evaluated using the proportion of participants who completed the study relative to the number of randomized participants. Adherence to the remote consumption monitoring was evaluated using the proportion of daily consumption texts received. We explored the variables that impacted adherence to remote reporting (i.e., predicted missing daily consumption text) by performing an exhaustive search of the model space^36^. The predictors included in the model selection were group, week, group and week interaction, and demographics of age, sex, race, education, income, and CUD. The optimal model was the one with the lowest Bayesian Information Criteria (BIC).

DD rates were calculated using Eq. 1^37^, where V is the indifference point between the smaller-sooner and larger-later rewards, A is the amount of the larger-later, *k* is the discounting rate, and D is the delay. DD rates (*k*) were natural-logarithmically transformed (ln(*k*)) to fulfill the normality assumption. A linear mixed-effects model including fixed effects of group (EFT/CET), session (S1-S5), and group and session interaction, and a random intercept of participants was conducted using ln(*k*) as outcome. The model was adjusted for covariates.


1 $$V=\frac{a}{1+\text{kD}}$$

Responses to the CPT were screened for overconsumption. As done previously, overconsumption was defined as purchasing more than 50g of cocaine at a given price for consumption over 24h^24^. Therefore, consumption during the CPT was capped at 50g, such purchases of more than 50g at any given price were transformed to 50g (n = 52 observations). Responses to the CPT were used to estimate intensity (i.e., consumption at price $0), alpha (i.e., elasticity of demand), O_max_ (i.e., maximum expenditure), and Pmax (i.e., price at which Omax occurs) using the *beezdemand* package^38^. Alpha was estimated based on fits of Eq. 2^39^ to the data, where Q is the consumption at price C, Q_0_ estimates consumption at price $0, α is the elasticity of demand, and k is the span of the function.


2 $$Q=Q*10^{k(e^{-aQ_{0}C}-1)}$$

Separate generalized linear mixed-effects models including fixed effects of group (EFT/CET), session (S1-S5), and group and session interaction, and a random intercept of participants were conducted using each demand parameter (intensity, alpha, O_max_, and P_max_) as outcomes. All models were adjusted for covariates. Alpha was natural-logarithmically transformed to fulfill the normality assumption. Data for which the equation did not converge were excluded for alpha analysis (n = 1 in S1; n = 8 in S2; n = 1 in S3; n = 7 in S4; n = 10 in S5).

Changes in daily cocaine consumption were analyzed using both a continuous (0–4 scale) and binary (use vs. no use) outcome. As part of our data integrity assessment, we evaluated the association between the self-reports of cocaine use and the urinalysis results. Additionally, to corroborate our scale measure, we tested the correlation between the daily scale score and the daily grams consumed on the subset of participants who reported daily grams (EFT = 29, CET = 29). Separate generalized linear mixed-effects models including fixed effects of group (EFT/CET), week (Weeks1–5), and group and week interaction, and a random intercept of participants were conducted for each outcome (continuous and binary). Both models were adjusted for covariates.

## Results

A total of 139 participants provided informed consent, and 113 (excluding the six participants matched to their partners) were randomized to EFT (n = 56) or CET (n = 57). Of those, 107 started the intervention (i.e., underwent the first cue generation; EFT = 54, CET = 53), and 85 completed the entire study. Reasons for discontinuation during intervention included voluntary withdrawal and loss to follow-up (i.e., stopping communication with the study team). After data cleaning, the final analytical sample for the complete case analysis included 80 participants (EFT = 41, CET = 39). Recruitment and data collection occurred between March/2023 and November/2025.

Table 1 summarizes baseline characteristics for EFT and CET participants in the complete case sample (see **Table S3** for intention-to-treat sample). Groups were well balanced across demographic measures and randomization variables (i.e., ln(k) and sex). A significant difference between groups was only observed for employment (*p*=.005), with EFT having more unemployed participants than CET.

Table 2 shows study retention and adherence to daily consumption report rates for both groups (see **Table S4** for retention and adherence for the intention-to-treat sample). Completion rate was above 70% in both groups, with higher rates for EFT than for CET. Daily consumption submissions were above 80% for both groups, and submission rates were comparable between groups across monitoring phases. Model selection results indicated that phase was the best predictor of missing daily consumption. Specifically, the model indicated that adherence improved with time in the study and participants were less likely to miss a submission during Weeks 2–4 relative to Week 1 (*p*s<.018; see **Table S5** for model estimates). No significant differences were observed between Week 5 and Week 1 (*p*=.171). Group was not included in the optimal model, indicating group differences in daily consumption submissions were not supported by the data. Similar findings were observed in the intention-to-treat sample (see **Table S5**), except that the model indicated significantly more submissions were missing in Week 5 than in Week 1 (*p*=.004).

Figure 2 shows ln(*k*) across sessions for both groups. Results from the linear mixed-effects model for ln(k) indicated a significant Group by Session interaction (*p*=.006), indicating that ln(k) differed between groups across sessions. The fixed effects of Session (*p*=.676) and Group (*p*=.156) were not significant (see **Table S6** for model estimates). Simple interaction contrasts indicated that participants in the EFT group showed greater reductions in ln(k) from baseline (S1) to all intervention sessions (S2-S5; *p*s≤.005) than CET. For the EFT group, pairwise comparisons indicated that ln(k) at each intervention session (S2-S5) was significantly lower than at baseline (S1; *p*s≤.002), but not different from each other (*p*s>.570). For CET, ln(k) did not change significantly across sessions (*p*s>.703). These findings were replicated in the intention-to-treat sample (see **Table S6**).

The generalized mixed-effects model for intensity of demand revealed a significant fixed effect of Session (*p*=.025). Neither the fixed effect of Group (*p*=.660) nor Group by Session interaction (*p*=.162) were significant (see **Table S7**). Pairwise comparisons did not identify any significant differences between individual sessions (*p*s>.078). Similar findings were observed in the intention-to-treat sample (see **Table S7**), except that pairwise comparisons indicated higher intensity at S4 and S5 than at S2 (*p*s<.019). The generalized mixed-effects model for alpha revealed a significant Group by Session interaction (*p*=.005), indicating that alpha differed between groups across sessions. The fixed effects of Session (*p*=.154) and Group (*p*=.830) were not significant (see **Table S8)**. Simple interaction contrasts indicated no significant differences in changes in alpha from baseline (S1) to the intervention sessions (S2-S5; *p*s>.310) between groups. Pairwise comparisons indicated that for EFT, alpha was significantly higher at S3 relative to S1, S2 (*p*s<.005). Significant differences were not observed between any other sessions (*p*s>.054). For CET, significant differences were not observed between any sessions (*p*s>.153). In the model for alpha on the intention-to-treat sample, only the fixed effect of Session was significant (see **Table S8**). The generalized mixed-effects models for O_max_, and P_max_ revealed no significant main effects of Session, Group, or Group by Session interaction. The O_max_ findings were replicated in the intention-to-treat sample. The intention-to-treat model on P_max_ revealed significant fixed effects of Session *p*=.023) and Group (*p*=.008), but no significant Group by Session interaction (*p*=.326). See **Tables S9-S10** for the model estimates for each outcome.

Figure 3 shows average daily cocaine consumption (on a scale from 0 = none to 4 = maximum ever used) across study weeks for both groups. Results from the linear mixed-effects model on the daily scale of cocaine consumption revealed a significant fixed effect of Week (p=.024) and a Group by Week interaction (*p*<.001), indicating that cocaine consumption differed between the groups across weeks. The fixed effect of Group (*p*=.989) was not significant (see **Table S11** for model estimates). Simple interaction contrasts indicated that participants in the EFT group showed greater reduction in cocaine consumption from Week 1 to all intervention weeks (Weeks 2–5; *p*s≤.036) than CET. Pairwise comparisons indicated that, for EFT, consumption was significantly lower during Weeks 3–5 relative to Week 1 (*p*s<.001) and significantly lower during Week 4 relative to Week 2 (*p*=.030). Significant differences were not observed during any other weeks (*p*s≥.064). For CET, consumption was significantly lower during Week 3 relative to Week 2 (*p*=.017) but not different across any other weeks (*p*s>.100). Notably, daily scale scores were significantly correlated with the number of grams reported (r = 0.68, p<.001). These findings were replicated in the intention-to-treat sample (see **Table S11**).

Results from the mixed-effects model on the binary daily consumption outcome (use vs. no use) indicated no significant main effects of Group (*p*=.590), Week (p=.173), or Group by Week interaction (*p*=.301; see **Table S12** for model estimates). Thus, no significant differences in frequency of use were observed between groups or across weeks. Results from urinalysis were consistent with self-reports (see **Table S13**). These results suggest that EFT effectively decreased the amount of cocaine consumed daily. However, significant changes in consumption frequency were not observed. Similar findings were observed in the intention-to-treat sample (see **Tables S12 and S14**).

## Discussion

The present pilot trial aimed to evaluate the: 1) feasibility of prolonged EFT exposure and remote monitoring of cocaine consumption; 2) efficacy of prolonged exposure to EFT in decreasing DD and cocaine consumption; and 3) efficacy of prolonged exposure to EFT in decreasing hypothetical demand for cocaine. We demonstrated that prolonged exposure to daily EFT reminders and daily consumption reporting was feasible, with more than 70% of participants in both groups completing the study. Additionally, consistent with our hypotheses, significant decreases in DD and amount of cocaine consumption were observed for participants in the EFT group, but not in CET. However, contrary to our hypothesis, robust effects of EFT on hypothetical demand were not observed.

First, consistent with our hypothesis, retention or adherence rates were overall high and similar between groups, suggesting that an intervention comprising multiple cue-generation sessions and several weeks of daily cue reminders and remote consumption reports is feasible. These findings are promising, especially given that high dropout rates are common in CUD studies and treatments. For example, a recent review found that attrition rates exceeding 50% are not uncommon in CUD treatment studies^40^. Several factors may have contributed to our retention and adherence success. For example, to increase rapport between participants and the study team, we ensured that each participant worked with and communicated with the same research assistant throughout the study. Additionally, daily reminders and consumption reports were delivered via text messages sent directly by the research team. This decreased any technological barriers for the participant to engage with study procedures.

Second, we replicated previous findings by showing significant decreases in DD following EFT but not CET. The effects of EFT on DD among individuals with CUD have been previously demonstrated^23,24^; however, in those studies the effects of EFT were evaluated following a single cue-generation session. Here, we extend these findings by showing a significant reduction in DD over a 4-week intervention, during which participants completed four cue-generation sessions and were exposed daily to their cue reminders.

Additionally, we contribute to the literature on the effects of EFT on real-world consumption. Previous studies have shown that EFT decreases real-world alcohol consumption^29,30^; however, no studies have examined EFT effects on real-world cocaine consumption. Here we showed that daily exposure to EFT significantly decreased the amount of cocaine consumed, relative to baseline. However, frequency of consumption did not seem to decrease with exposure to EFT. Therefore, it seems that among individuals with CUD, this length of EFT exposure might function as a harm reduction intervention rather than an intervention to sustain abstinence. Longer EFT exposure might be needed to sufficiently reduce consumption frequency. Indeed, the intervention duration in our current pilot trial (4 weeks) was shorter than the typical duration for CUD trials (6–36 weeks^41^). Furthermore, evidence suggests that intensive treatment (i.e., several hours/week over the course of many weeks) is commonly needed to reduce cocaine use^7^ and that most psychosocial interventions (with the exception of contingency management) are not effective in increasing the likelihood of negative urinalysis^42^.

Lastly, contrary to our hypothesis, we did not see any robust effects of EFT on behavioral economics demand parameters (i.e., intensity, alpha, O_max_, and P_max_). Although EFT showed a significant increase in alpha (i.e., increase in sensitivity to price) during the second intervention session (S3) relative to baseline (S1), this increase was not sustained across the remaining sessions. Furthermore, simple interaction contrast did not reveal any significant differences in change from baseline between groups. Similarly, no significant interactions were observed for the other demand parameters. The reason for the lack of significant demand effects is unclear. First, changes in the CPT task midway through the study resulted in a smaller sample size for the CPT, which likely compromised our ability to detect changes in hypothetical demand for cocaine. Another possible explanation for the lack of effect in the present study could be that EFT did not meaningfully impact cocaine valuation. Indeed, although we saw a decrease in amount consumed, we did not see a significant decrease in frequency of use, which could indicate that cocaine still held high value among the sample. Conversely, if the decrease in amount consumed is associated with decreases in cocaine valuation, this decrease could have been marginal, and our hypothetical task might not be sensitive enough to capture small changes in value. Notably, decreases in substance consumption without concomitant changes in demand following EFT have been previously reported^29^. Thus, more studies are needed to investigate the optimal conditions under which EFT affects demand parameters.

Some limitations are noteworthy. First, participants were exposed to their cues twice a day at fixed times. However, it is currently unknown which is the optimal frequency of reminder delivery for this population, and whether the timing of the reminders aligned with peak craving or consumption. For example, cue exposure at different times (e.g., closer to the time of consumption) could have produced more robust effects of EFT on cocaine use. Furthermore, we were unable to measure the extent to which participants actually engaged with their cues (e.g., how many minutes they spent reading and thinking about them daily). Another limitation is that our biochemical verification of cocaine use (urinalysis) was not sensitive to changes in quantity of use but only detected use or no use. Thus, we had no biochemical verification of the accuracy of self-reports. Furthermore, cocaine can show in urinalysis for more than 14 days after the last dose for individuals who use heavily^43^. Thus, we would be unlikely to detect changes in the frequency of use with our frequent urinalysis screening. Lastly, our study did not include any follow-up measures. Thus, it is unclear whether the effects observed persisted after the intervention was discontinued.

Future studies exploring whether the effects of EFT on cocaine consumption are long-lasting are still needed. Studies exploring how to optimize EFT delivery to result in more robust effects on cocaine consumption are also warranted. Lastly, further investigation into the conditions under which EFT impacts hypothetical demand and into how the observed consumption effects relate to changes in cocaine valuation is necessary to provide further support for the reinforcer pathology theory, which treats demand as one of its main measures of reinforcer valuation.

Overall, the present findings add to the current literature by replicating and extending the effects of EFT on DD among individuals with CUD and by being the first study to demonstrate that EFT decreases the amount of daily cocaine use. Our results suggest that EFT might be a promising intervention for harm reduction among individuals who use cocaine. EFT could also be used to augment other interventions for CUD by contributing to significant decreases in daily consumption until abstinence is achieved. EFT presents the advantage of being easy to implement and scale and can also be used for participants who are non-treatment-seeking, given that it is not an intervention focused on cocaine use. However, studies exploring the optimal conditions under which EFT produces significant and long-lasting effects on cocaine use are still needed.

## Supplementary Material

Supplementary Files

This is a list of supplementary files associated with this preprint. Click to download.

- SupplementalMaterials.docx

## Figures and Tables

**Figure 1:**
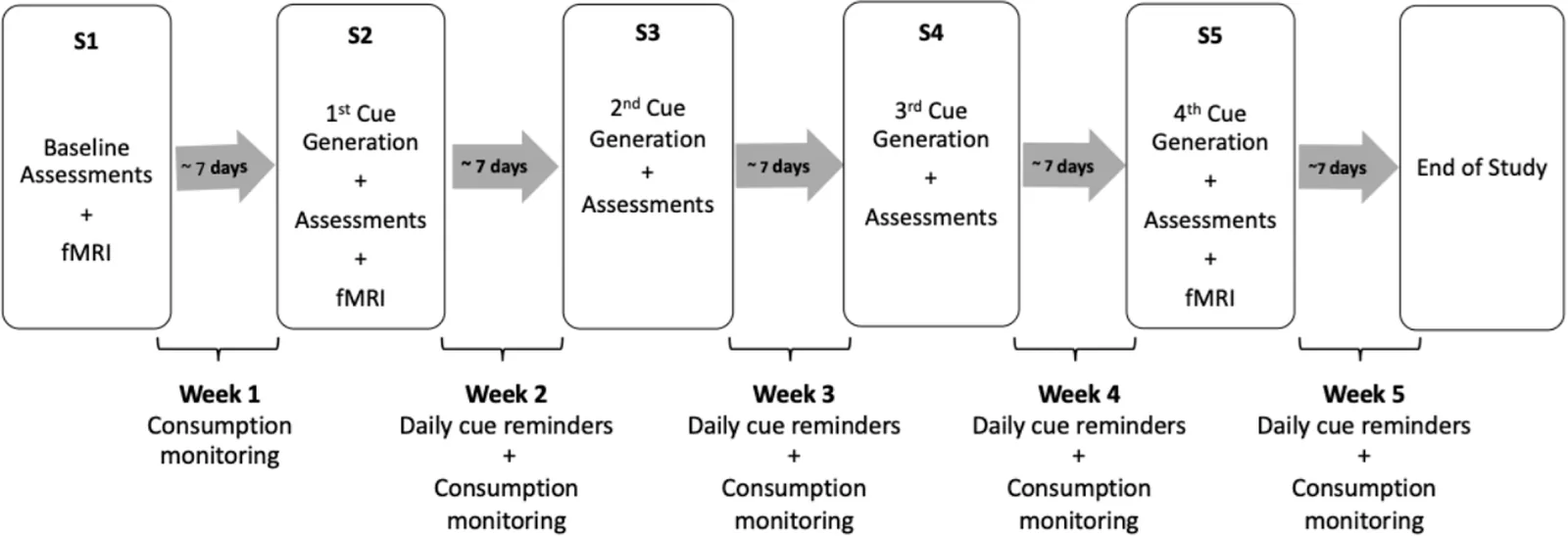
Study timeline.

**Figure 2:**
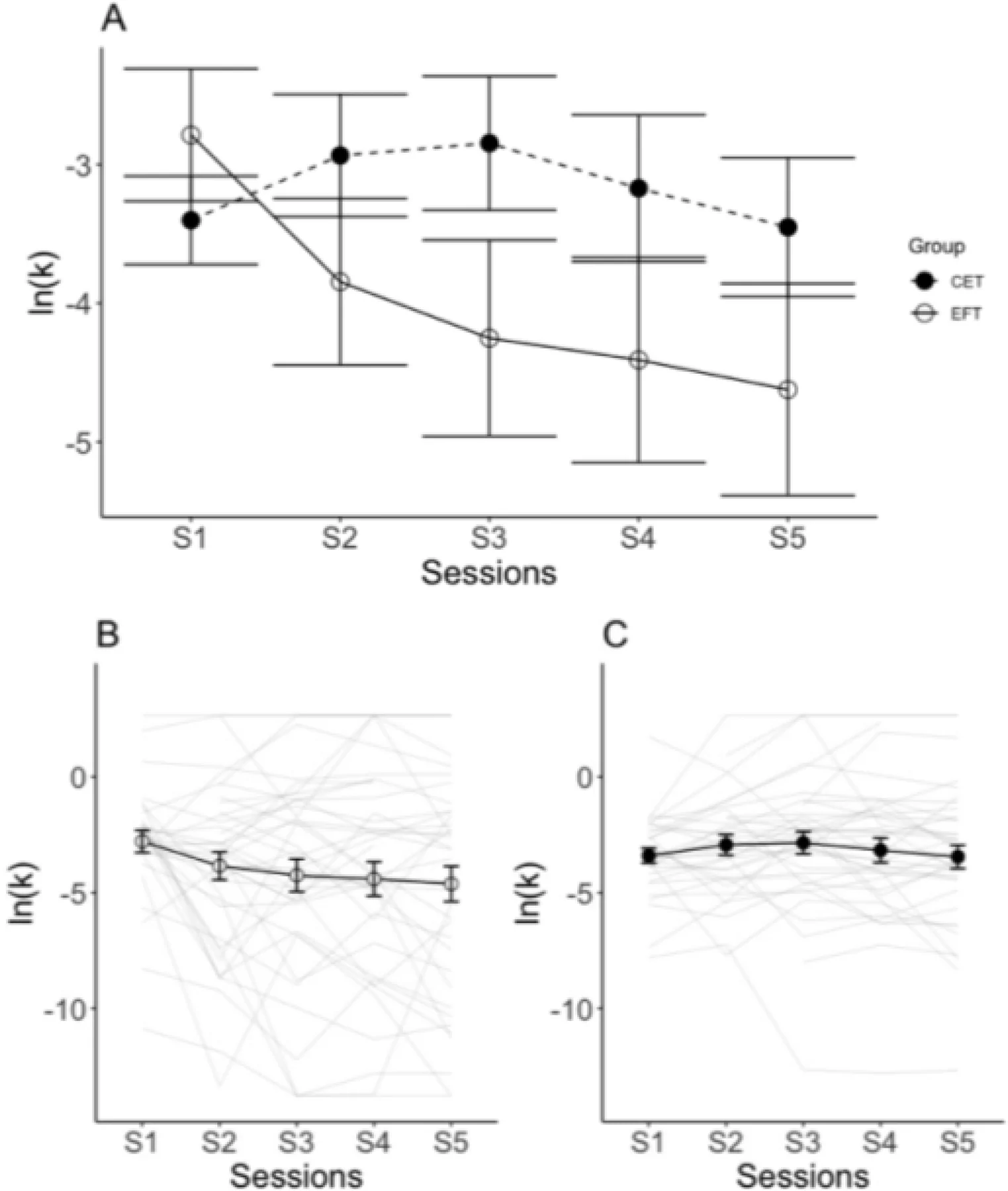
**A)** Average discounting rates across sessions for EFT and CET. **B)** Discounting rates across sessions for EFT. Grey lines represent individual data, and open circles represent the group average. **C)** Discounting rates across sessions for CET. Grey lines represent individual data, and filled circles represent the group average. In all panels, error bars represent the standard error of the mean (SEM).

**Figure 3:**
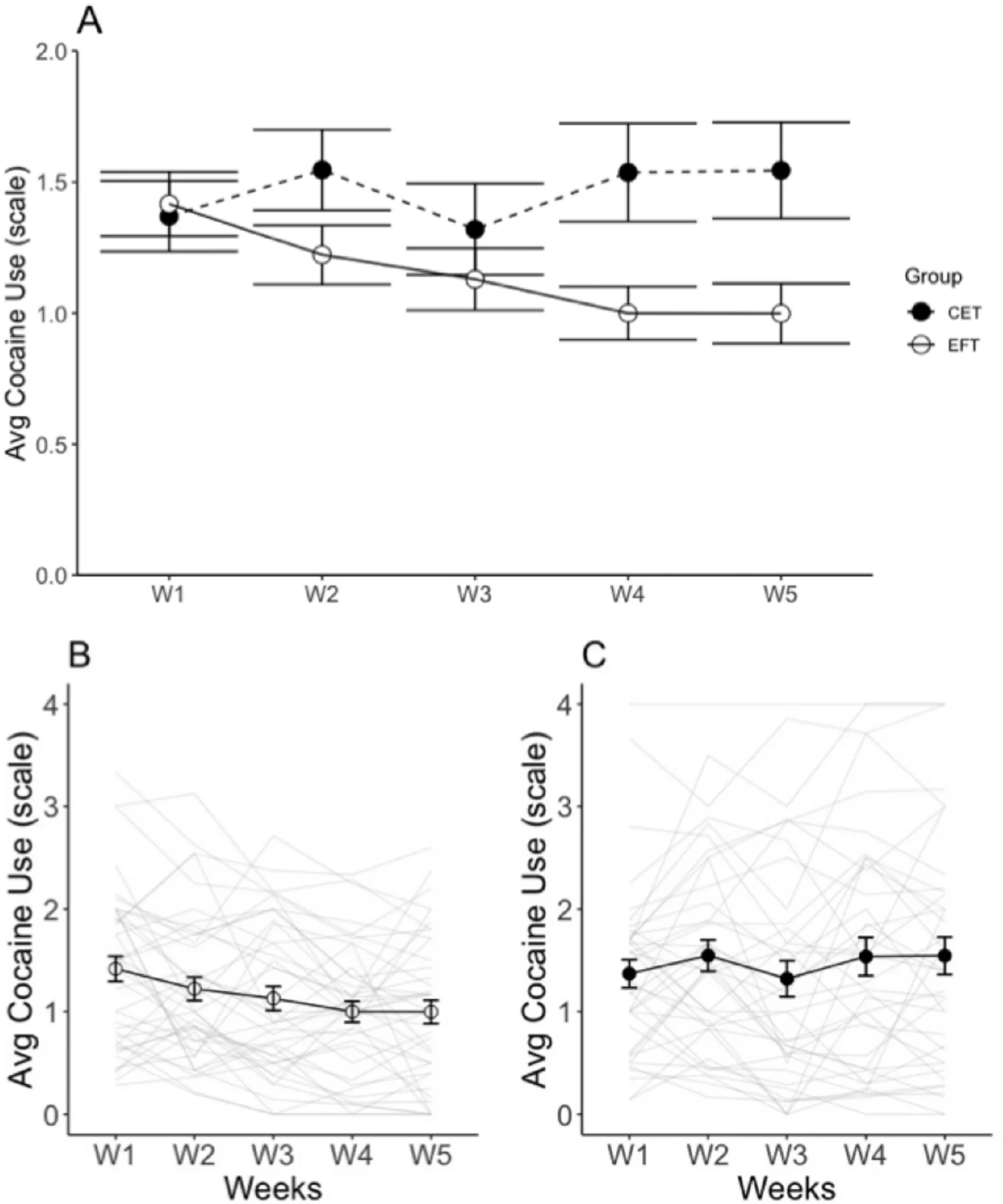
**A)** Average cocaine consumption (on a scale from 0=none to 4=maximum ever used) across monitoring phases for EFT and CET. **B)** Drinks/day across monitoring phases for EFT. Grey lines represent individual data, and open circles represent the group average. **C)** Drinks/day across monitoring phases for CET. Grey lines represent individual data, and filled circles represent the group average. In all panels, error bars represent the standard error of the mean (SEM).

**Table 1:** Sample demographics.

Variable	EFT	CET	*p*-value
n	41	39	
Age (mean (SD))	46.73 (10.50)	44.64 (10.67)	.380
Sex = Male (%)	18 (43.9)	22 (56.4)	.371
Ethnicity (%)			.586
Hispanic or Latino	1 (2.4)	1 (2.6)	
Not Hispanic or Latino	40 (97.6)	37 (94.9)	
Prefer not to answer	0 (0.0)	1 (2.6)	
Race (%)			.500
American Indian/Alaska Native	0 (0.0)	1 (2.6)	
Black or African American	16 (39.0)	19 (48.7)	
More Than One Race	3 (7.3)	2 (5.1)	
White	22 (53.7)	16 (41.0)	
Prefer not to answer	0 (0.0)	1 (2.6)	
Education (%)			.591
High School (9 + years)	24 (58.5)	27 (69.2)	
College (13 + years)	16 (39.0)	11 (28.2)	
Graduate School (17 + years)	1 (2.4)	1 (2.6)	
Employment (%)			.005
Part time	14 (34.1)	2 (5.1)	
Full time	4 (9.8)	4 (10.3)	
Unemployed	23 (56.1)	33 (84.6)	
Income (%)			.815
No income	11 (26.8)	13 (33.3)	
Less than $1,000	25 (61.0)	21 (53.8)	
$2,000 through $2,999	4 (9.8)	3 (7.7)	
$3,000 or more	1 (2.4)	2 (5.1)	
SUD criteria (mean (SD))			
Cocaine	9.20 (2.35)	8.87 (2.73)	.571
Alcohol	2.78 (4.16)	2.73 (3.68)	.953
Cannabis	2.07 (3.35)	2.13 (3.59)	.944
Stimulants	1.73 (3.86)	0.56 (1.94)	.094
Opioids	1.15 (3.05)	1.23 (3.29)	.906
Tobacco	3.37 (3.66)	3.23 (3.61)	.868
ln(*k*)	−2.79 (2.61)	−3.40 (1.77)	.286
Baseline Cocaine Consumption (scale)	1.45 (1.27)	1.28 (1.35)	.093

*Note*. SUD = substance use disorder

**Table 2:** Retention and adherence rates

Retention rates	EFT	CET
	77.3%	72.2%
Daily submission rates		
Week 1	83.9%	89.0%
Week 2	95.3%	91.5%
Week 3	91.7%	97.8%
Week 4	91.4%	90.0%
Week 5	86.1%	80.0%

*Note*. Retention rate calculated based on the participants included in the complete case analysis (107 independently randomized [EFT = 53, CET = 54], 80 completers [EFT = 41, CET = 39]). Submission rates were calculated using the 80 participants in the complete-case sample. Retention and submission rates for the intention-to-treat sample (n = 119) can be found in **Table S6** in the Supplemental Materials.
